# Torsional and lateral eigenmode oscillations for atomic resolution imaging of HOPG in air under ambient conditions

**DOI:** 10.1038/s41598-022-13065-9

**Published:** 2022-05-28

**Authors:** Anna L. Eichhorn, Christian Dietz

**Affiliations:** Physics of Surfaces, Institute of Materials Science, Technische Universität Darmstadt, Alarich-Weiss-Str. 2, 64287 Darmstadt, Germany

**Keywords:** Graphene, Nanoscale materials

## Abstract

Combined in-plane and out-of-plane multifrequency atomic force microscopy techniques have been demonstrated to be important tools to decipher spatial differences of sample surfaces at the atomic scale. The analysis of physical properties perpendicular to the sample surface is routinely achieved from flexural cantilever oscillations, whereas the interpretation of in-plane sample properties via force microscopy is still challenging. Besides the torsional oscillation, there is the additional option to exploit the lateral oscillation of the cantilever for in-plane surface analysis. In this study, we used different multifrequency force microscopy approaches to attain better understanding of the interactions between a super-sharp tip and an HOPG surface focusing on the discrimination between friction and shear forces. We found that the lateral eigenmode is suitable for the determination of the shear modulus whereas the torsional eigenmode provides information on local friction forces between tip and sample. Based on the results, we propose that the full set of elastic constants of graphite can be determined from combined in-plane and out-of-plane multifrequency atomic force microscopy if ultrasmall amplitudes and high force constants are used.

## Introduction

Understanding the in-plane nanomechanical behavior of graphitic surfaces at the atomic scale under ambient conditions in air is of utmost importance to predict the long-term performance of graphene-based nanodevices such as van der Waals heterostructures^1^ or for DNA sequencing^2^. Multifrequency atomic force microscopy (AFM)^3^ was shown to be an excellent tool for the quantification of forces at the nanoscale in out-of-plane as well as in in-plane direction^4–6^. The out-of-plane force deconvolution and determination of elastic moduli from bimodal AFM spectroscopy data using the Sader method^7^ or the matrix method^8^ are well established. In contrast, the determination of in-plane forces and shear moduli from spectroscopic data is still challenging^9,10^. One issue is the difficulty to excite the cantilever in the in-plane direction. This problem can either be addressed by using qPlus sensors^11–13^ or by photothermal excitation^14,15^. The photothermal excitation technique is based on a power-modulated laser focused at the base of the cantilever and a few micrometers off the cantilever length symmetry axis. This results in an in-plane oscillation if the excitation frequency matches the resonance frequency of the desired eigenmode. Photothermal excitation facilitated atomic resolution imaging using the torsional-eigenmode oscillation in bimodal AFM^16^. A second issue is the discrimination between torsional and lateral eigenmodes. In most publications the term “lateral” is used as synonym for both, the torsional and lateral eigenmode. This might originate from the fact that in theory the lateral-eigenmode oscillation should not be observable using the beam detection methods as schematically explained by Ding et al*.*^17^ and illustrated in Fig. S1. However, they rationalized that the tip attached to the cantilever can induce coupling between lateral and torsional eigenmodes facilitating the detection of the lateral resonance using a standard detection laser and segmented photodiodes. The authors demonstrated that both eigenmodes are useful for imaging and that the lateral oscillation might be advantageous over the torsional one. The main issue in using the lateral eigenmode for imaging is the calibration of the inverse optical lever sensitivity (invOLS) to attain quantitative or semi-quantitative data. There are a few approaches for the determination of the torsional invOLS^18–22^, whereas there is currently no procedure for the determination of the lateral invOLS to the best of our knowledge.

In this study, we compared the suitability of the torsional and the lateral eigenmodes for atomic resolution imaging of HOPG in air under ambient conditions. The comparison is based on our recently published methodology named AMFlex2-FMTor1-FMFlex3 mode introduced in Ref.^4^ with a slightly modified setup (AMFlex2-FMLat1-FMFlex3). Both setups exploit the second flexural eigenmode for the topographical feedback in amplitude modulation (AM) whereas the first torsional, first lateral and third flexural mode are frequency-modulated (FM) and controlled by phase-locked-loop (PLL) electronics. Inspired by the work of colleagues in the field, we used higher flexural eigenmodes which were shown to be beneficial for atomic resolution imaging due to the enhanced stiffness compared to the first flexural eigenmode^23,24^. Additionally, the combination of small free amplitudes and small amplitude setpoints was used for imaging as it was shown by Santos et al*.*^25,26^ and Lai et al*.*^27^ that the method can lead to a very close proximity between tip and sample, being essential for high resolution imaging.

**Figure 1:**
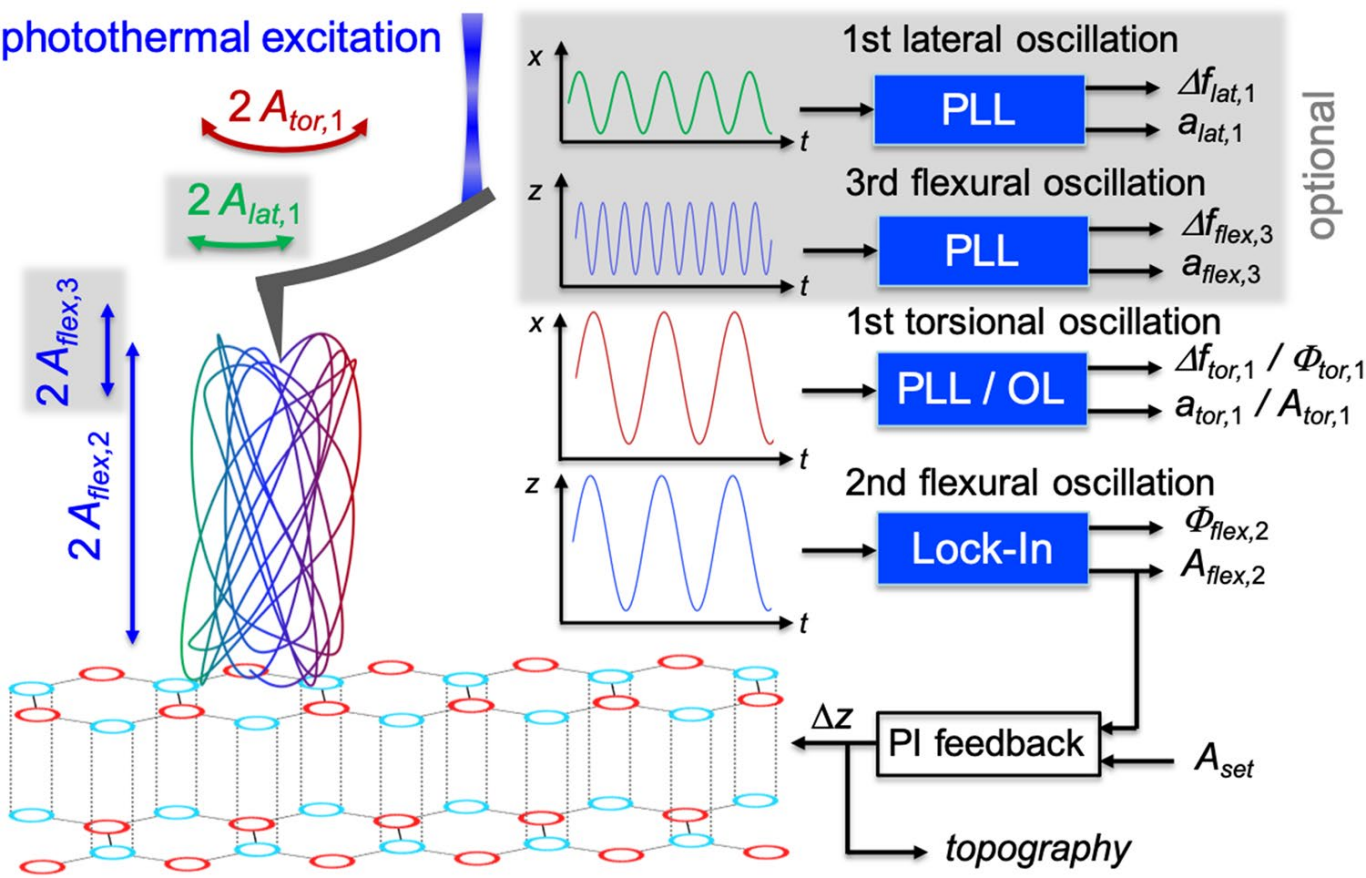
Schematic illustration depicting the setup of the AMFlex2-OLTor1-FMLat1-FMFlex3 mode. Topographical feedback is driven at the coupled resonance frequency of the second flexural and the first torsional eigenmode using the flexural component for amplitude modulation. The first-torsional-eigenmode amplitude and phase are recorded at the same frequency in an open-loop configuration. Optionally, the setup can be expanded by one or two phase-locked loops, tracking the phase at resonance of the first lateral and/or the third flexural eigenmode which allowed us to quantify forces in in-plane and/or out-of-plane direction from the respective frequency-shift data. Dissipative tip-sample interactions can be analyzed from the drive amplitudes (*a*_*lat,*1_, *a*_*flex*,3_) and the respective phase shifts (*Φ*_*flex*,2_, *Φ*_*tor,*1_). The cantilever dimension was substantially reduced for simplicity.

For some of the super-sharp tips we used, it was impossible to excite the second flexural and the first torsional eigenmode individually due to the close proximity of their resonance frequencies. Interestingly, the coupling of the two eigenmodes facilitated atomic resolution imaging. A scheme of possible setups as well as the resulting movement of the tip are schematically shown in Fig. 1. If there is coupling between the second flexural and the first torsional eigenmode, the torsional oscillation cannot be controlled individually (open loop (OL)). From the second flexural and the first torsional phase shifts, dissipation in out-of-plane and in-plane direction can be determined, respectively. Unfortunately, the AMFlex2-OLTor1 mode is not suitable for the deconvolution of in-plane or out-of-plane forces. Consequently, we extended the AMFlex2-OLTor1 mode, by two phase-locked loops (PLL) for frequency modulation (FM) of the first lateral and the third flexural eigenmode. The resulting AMFlex2-OLTor1-FMLat1-FMFlex3 mode such as schematically depicted in Fig. 1 facilitates quantification of in-plane and out-of-plane forces, reconstructed from lateral- and third-eigenmode flexural frequency-shift data.

In this work, we analyzed the imaging capability of coupled flexural/torsional oscillations as well as the suitability of the first lateral eigenmode for atomic resolution imaging and provide an approach for the calibration of the lateral eigenmode optical lever sensitivity. In addition, we calculated in-plane forces from torsional and lateral frequency-shift data and analyzed the origin of the forces regarding friction or shear. Analyzing the tip-trajectories, effective torsional and lateral amplitudes as well as the maximum indentation of the tip into the surface (with and without compression) were estimated. The plausibility of the determined lateral forces was confirmed by calculating the lateral displacement using the literature value of the shear modulus of HOPG.

## Results and discussion

### Imaging capability of the AMFlex2-OLTor1-FMLat1 mode for atomic resolution

**Figure 2:**
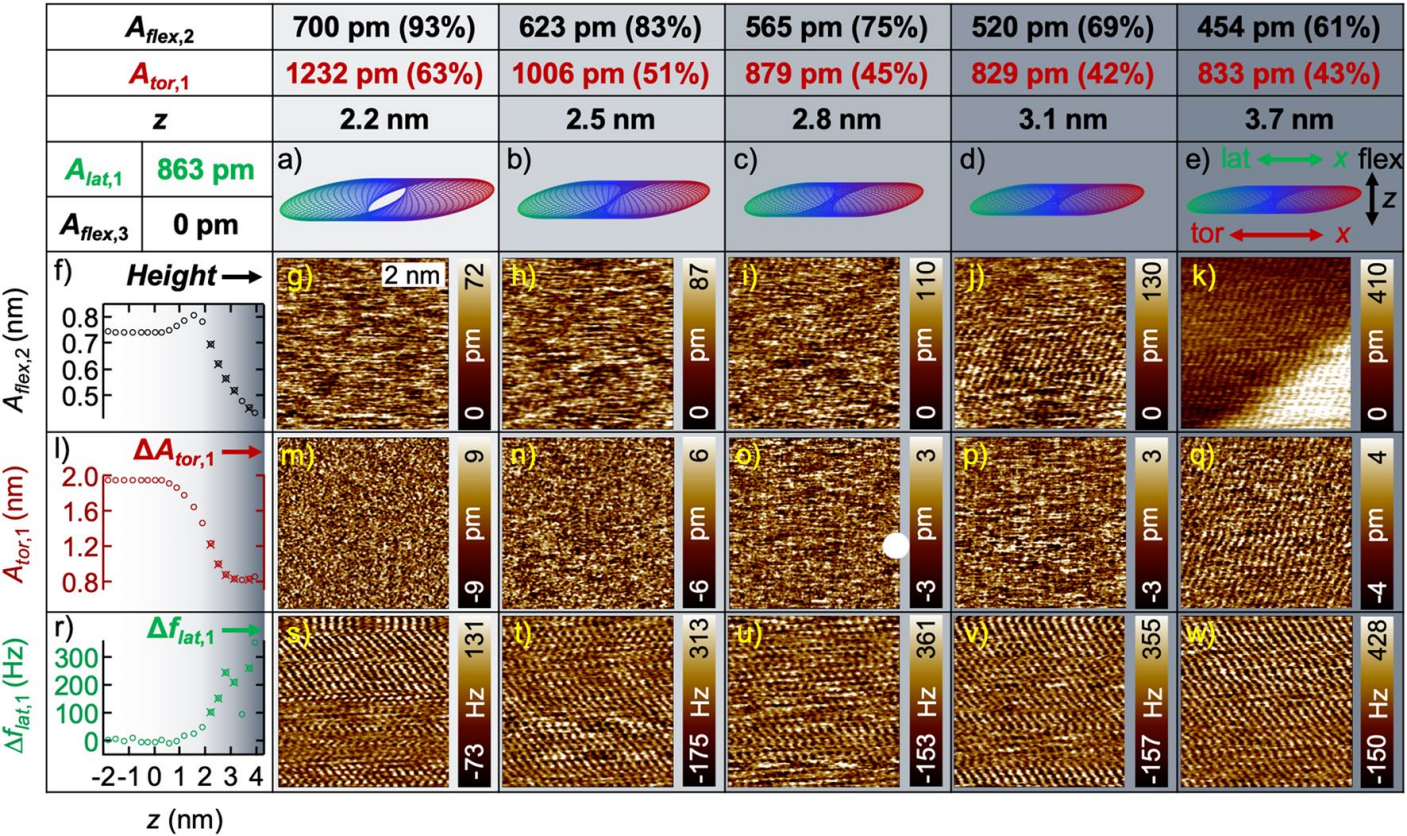
Imaging and spectroscopy on HOPG at a covered step edge in AMFlex2-OLTor1-FMLat1 mode with 863 pm lateral-eigenmode-amplitude setpoint. (**a**–**e**) Schemes of the tip trajectory for the coupled second flexural and first torsional cantilever oscillation for decreasing second flexural-eigenmode-amplitude setpoints *A*_*flex*,2_. The setpoints of *A*_*flex*,2_ are listed in the first, the corresponding setpoints of the first-torsional-eigenmode amplitude *A*_*tor*,1_ in the second row and the approximate *z*-sensor position at the third row. The values in brackets represent the percentages of the setpoints from the free amplitudes *A*_0*(flex*,2*)*_ and *A*_0*(tor*,1*)*_. (**f**) Second flexural-, (**l**) first torsional-eigenmode amplitude *A*_*tor*,1_ and (**r**) first lateral frequency shift Δ*f*_*lat*,1_ *vs. z*-sensor position, where the crossed circles mark the positions at which the images where taken. (**g**–**k**) Height, (**m**–**q**) first torsional-eigenmode amplitude error Δ*A*_*tor*,1_ and (**s**–**w**) Δ*f*_*lat*,1_ images at decreasing *A*_*flex*,2_. Note, that *Φ*_*tor,*1_ was assumed to be 90° for the calculation of the tip-trajectories shown in (**a**–**e**).

From Fig. 2, we get an overview of the capability of different imaging channels for atomic resolution imaging in air under ambient conditions depending on the *z*-sensor position. Increasing *z*-sensor position (from left to right) causes a reduction of the average tip-sample distance. Figure 2a–e show schemes of the expected tip-trajectory corresponding to the AMFlex2-OLTor1-FMLat1 mode with a lateral-eigenmode amplitude setpoint of *A*_*lat,*1_ = 863 pm. The shown tip-trajectories were calculated by plotting the out-of-plane deflection *z*(*t*) *vs.* the in-plane deflection *x*(*t*) of the cantilever using linear combinations of cosine functions such as shown by Benaglia et al*.*^28^ for the *z*-component and assuming that *Φ*_*tor,*1_ = 90° (see Eqs. (S1, S2) and Fig. S2 of the Supplementary Information for details). The lateral-eigenmode invOLS was calibrated by imaging a wrinkle of a graphene layer on an HOPG sample with different lateral amplitude setpoints while oscillating perpendicular to the wrinkle and comparing the images with the results of a Savitzky-Golay-filtered reference image. A detailed description of the technique can be found in Section 4 (Calibration of lateral-oscillation-eigenmode sensitivity) of the Supplementary Information. From Fig. 2a–e the second flexural-eigenmode-amplitude setpoint is reduced from 700 to 454 pm, causing a reduction of the torsional amplitude due to the coupling of both modes (Fig. 2, second row). The corresponding approximate *z*-sensor position can be taken from the third row of Fig. 2. From spectroscopic experiments (local amplitude distance curves recording all available observables), we can visualize the dependence of the second flexural amplitude (Fig. 2f), the first torsional amplitude (Fig. 2l) and the first lateral frequency shift (Fig. 2r) on the *z*-sensor position. The crossed circles mark the positions where the AFM images of Fig. 2 where taken. The height images are shown in Fig. 2g–k, the “error images” of the first torsional amplitude in Fig. 2m–q and the first lateral frequency-shift images in Fig. 2s–w. “Error images” of the first torsional amplitude here means the deviation of the actual torsional amplitude relative to the torsional-amplitude setpoint corresponding to the second flexural-amplitude setpoint due to the coupling of both eigenmodes.

Comparing the amplitude *vs. z*-position curves in Fig. 2f,l, it becomes evident that although the second flexural and the first torsional oscillations are coupled, they show different dependencies on the tip-sample distance. These differences are consistent with results from uncoupled flexural–torsional AFM studies in open-loop configuration^16^. The lateral frequency shift *vs. z*-sensor position curve in Fig. 2r shows an increasing repulsive interaction with decreasing tip-sample distance. Atomic resolution imaging was feasible in all three channels as e.g. visible in Fig. 2k,q,w at a *z*-sensor position of approximately 3.7 nm. Interestingly, the lateral frequency-shift images show atomically resolved structures at setpoint ratios up to 93% of the second flexural-eigenmode amplitude as observable in Fig. 2s, which corresponds to a *z*-sensor value of approximately 2.2 nm. Compared to imaging with “uncoupled” cantilevers, where atomic resolution imaging was impossible to achieve using setpoint ratios larger than 15%, it is reasonable to believe that performing measurements with “coupled” cantilevers implies more gentle conditions, preserving the integrity of both, tip and sample. Note, that the torsional amplitude ratio was already at 63% which fits to our observation that the stiffness of the second flexural eigenmode was higher compared to the first torsional eigenmode stiffness for the type of cantilevers used in this study (see “Materials and methods” for details). At this position, however, neither in the height image (Fig. 2g) nor in the torsional-amplitude error image (Fig. 2m) atomic contrast could be resolved. Lowering the second flexural-eigenmode-amplitude setpoint, atomic contrast becomes more and more apparent in the height images (Fig. 2m–q). The growing corrugation amplitude (measured vertical distance between atomic and hollow side) is assumed to originate from an increase in the mean cantilever deflection^16,29,30^. Strikingly, the height image at a *z*-sensor position of 3.7 nm (Fig. 2k) shows a step which matches the height of individual graphene layers. This step is neither observable in the Δ*A*_*tor*,1_ image (Fig. 2q) nor in the Δ*f*_*lat*,1_ image at the same *z*-sensor position. Consequently, the topographic feedback seems to function extremely precise. Another interesting observation is, that the atomic structure at both, the lower and the upper plateau of the graphene step, exhibit the same arrangement of carbon atoms. This leads us to the conclusion that we imaged a monoatomic step edge covered by a small number of graphene layers which is in line with results shown by Abooalizadeh et al*.*^31^. From this observation the question arises why we were not able to resolve the step at smaller *z*-sensor positions. To answer the question, we exemplarily drew two cross sectional profiles through the height images shown in Fig. 2k,j (see Fig. S3 in the Supplementary Information). We assume that the visibility of the step edge depends on whether the average tip-sample force (represented by the mean deflection, averaged over several oscillation cycles) is predominantly attractive or repulsive. On the right side of Fig. S3, we schematically sketched the alleged interaction between tip and sample on a covered step edge. In the predominantly attractive regime, we assume that several graphene layers cover the step such as depicted in the top right part of Fig. S3. As discussed in our recent work and by others^4,32^, it is reasonable to believe that the topmost carbon layers are lifted by the attractive tip-sample interactions. Consequently, the tip will not sense the step edge. If imaging takes place in the predominantly repulsive regime, we assume that the graphene cover layers are tightly stretched over the step edge as sketched in the bottom right of Fig. S3.

From the imaging results shown in Fig. 2 we can conclude that the coupled motion of the cantilever (AMFlex2-OLTor1) seems to promote atomic resolution at higher setpoint ratios (*A*_*flex,*2_/*A*_0*(flex,*2*)*_) compared to uncoupled ones. This can, on the one hand, be attributed to the enhanced dynamic stiffness of the coupled flexural/torsional oscillation of the cantilever and, on the other hand, to the oval shape of the tip trajectory. This might lead to a reduced influence of the interactions far away from the center of the oscillation, because the tip will have the closest distance to the sample and therefore the highest interaction at the currently measured local position. Nevertheless, the largest drawback of imaging with coupled cantilever modes is that the relation between the flexural and the torsional amplitudes cannot be set individually. Additionally, it needs to be considered that the combined in-plane oscillation of torsional and lateral components needs to be analyzed in detail in order to get a better understanding regarding the interaction with the sample. An idea for future studies would be tailoring cantilevers e.g. by a controlled introduction of holes at different positions of the cantilever such as shown by Eslami et al*.*^33^, in order to analyze the influence of different resonance-frequency ratios.

### Comparison of torsional and lateral frequency-shift images as a function of the amplitude setpoints

**Figure 3:**
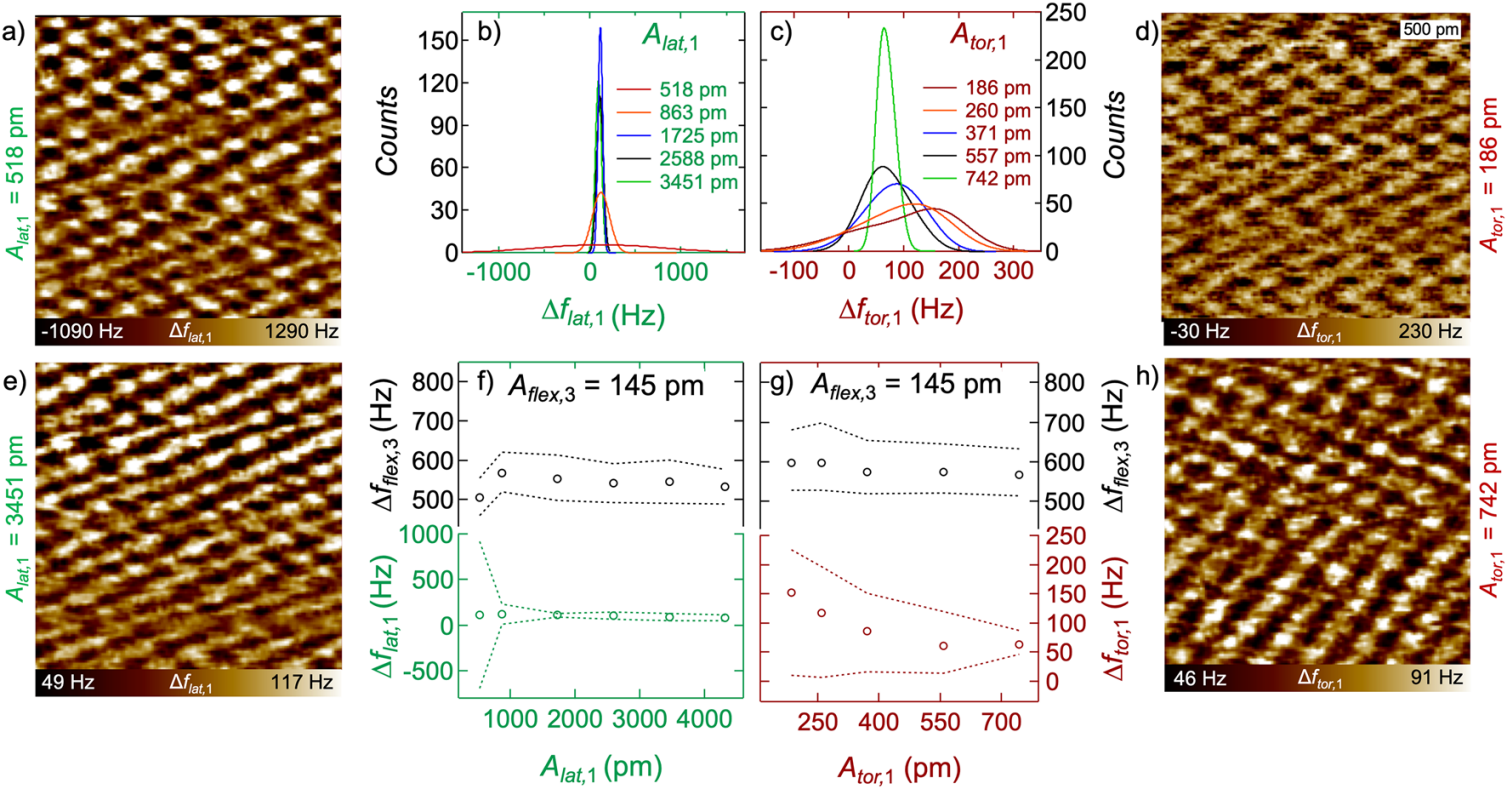
Influence of the lateral- and the torsional-amplitude setpoints on the frequency shifts in AMFlex2-FMLat1-FMFlex3 mode and in AMFlex2-FMTor1-FMFlex3 mode respectively. Frequency-shift images are shown exemplarily taken at *A*_*lat,*1_ = 518 pm in (**a**), *A*_*lat,*1_ = 3451 pm in (**e**) and *A*_*tor,*1_ = 158 pm in (**d**), *A*_*tor,*1_ = 628 pm in (**h**). Histograms of lateral (**b**) and torsional frequency-shift images (**c**) at different lateral-/torsional-amplitude setpoints. Third-eigenmode flexural and lateral (**f**)/torsional (**g**) frequency shifts at maximum counts (open circles) and at FWHM (dotted lines) as a function of the lateral-/torsional-amplitude setpoints at a constant third flexural-eigenmode amplitude of 145 pm and a constant second flexural-eigenmode amplitude of 110 pm.

In order to gain deeper insights into the different oscillation behaviors of the torsional and the lateral eigenmodes, we analyzed several 5 × 5 nm^2^ frequency-shift images taken in the AMFlex2-FMLat1-FMFlex3 mode and the AMFlex2-FMTor1-FMFlex3 mode at different lateral- and torsional-eigenmode setpoints. The results are shown in Fig. 3. In Fig. 3a,e and Fig. 3d,h lateral and torsional frequency-shift images (2.5 × 2.5 nm^2^ zoom-in) taken at *A*_*lat,*1_ = 518 pm (a), *A*_*lat,*1_ = 3451 pm (e) and *A*_*tor,*1_ = 158 pm (d), *A*_*tor,*1_ = 628 pm (h) are shown exemplarily. The corresponding histograms of the lateral and the torsional frequency-shift images can be seen in Fig. 3b,c, respectively. The open circles in Fig. 3f,g show the lateral and torsional frequency shifts at maximum counts and the dashed lines represent the full width at half maximum (FWHM) as a function of the lateral- and torsional-amplitude setpoints, respectively.

**Figure 4:**
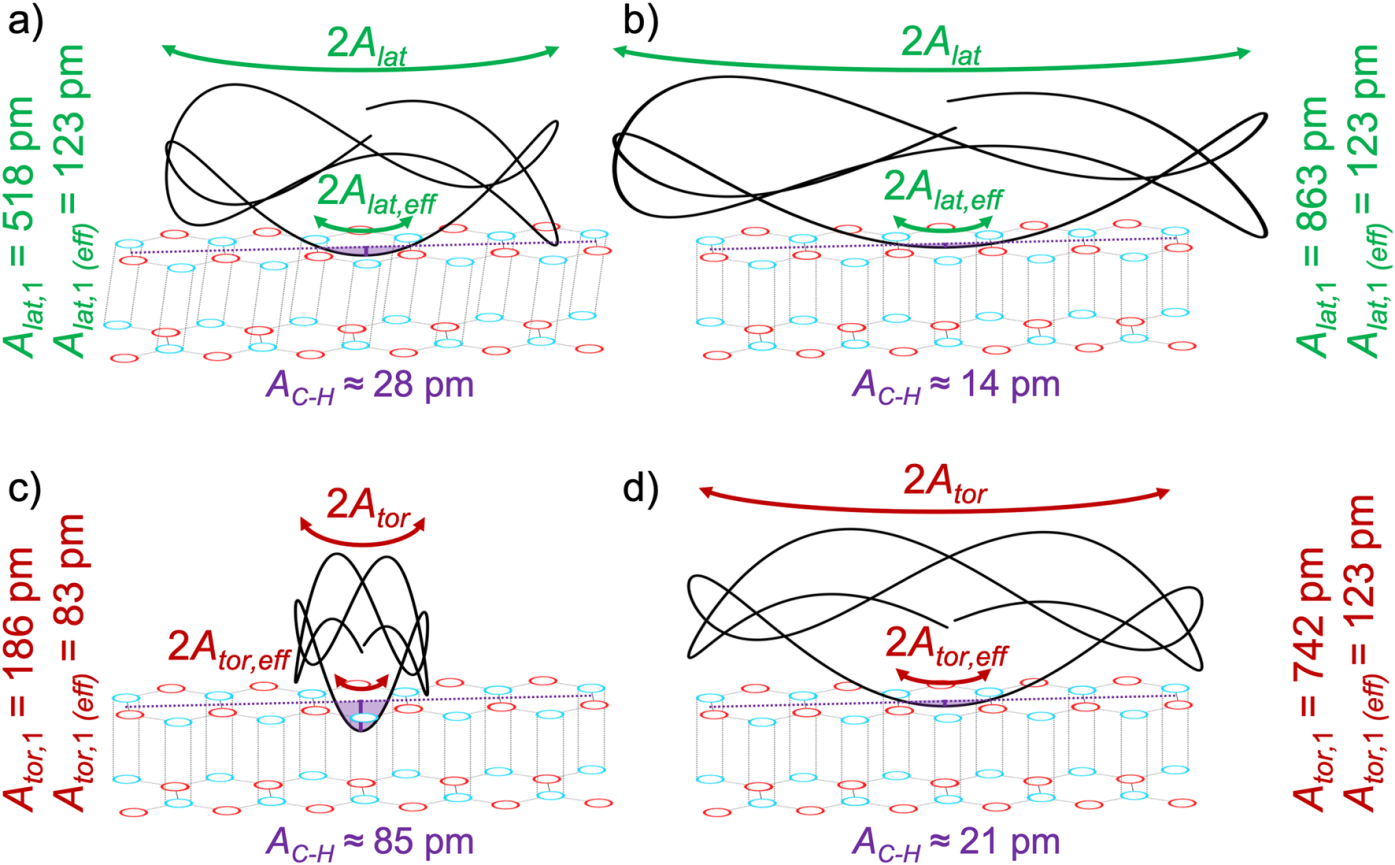
Extract of the calculated tip-trajectory of a cantilever oscillating in the AMFlex2-FMLat1-FMFlex3 mode (**a**,**b**) or in the AMFlex2-FMTor1-FMFlex3 mode (**c**,**d**) with *A*_*flex,*2_ = 110 pm and *A*_*flex,*3_ = 145 pm. The trajectories are shown for two different torsional- and lateral-oscillation eigenmodes: (**a**) *A*_*lat,*1_ = 518 pm, (**b**) *A*_*lat,*1_ = 863 pm, (**c**) *A*_*tor,*1_ = 186 pm and (**d**) *A*_*tor,*1_ = 742 pm. Additionally, the effective amplitudes (*A*_*lat,*1 *(eff)*_ and *A*_*tor,*1 *(eff)*_) as well as the determined corrugation amplitudes *A*_*C-H*_ are shown. The minimum possible distance between two hydrophobic surfaces (tip and sample) of 300 pm^34^ was neglected for the schematic drawing to improve the comprehensibility.

If we compare the frequency-shift images taken in the AMFlex2-FMLat1-FMFlex3 mode in Fig. 3a,e with the images taken in the AMFlex2-FMTor1-FMFlex3 mode in Fig. 3d,h we observe that atomic contrast was achieved in all images but the range of the frequency-shift values strongly differs. While the lateral and torsional frequency-shift images taken at the higher-amplitude setpoints (*A*_*lat,*1_ = 3451 pm and *A*_*tor,*1_ = 628 pm) shown in Fig. 3e,h show similar frequency-shift ranges, the frequency-shift images at smaller-amplitude setpoints (*A*_*lat,*1_ = 518 pm and *A*_*tor,*1_ = 158 pm) in Fig. 3a,e clearly differ regarding their frequency-shift ranges and the distribution of the values. This becomes even more evident by looking at the histograms corresponding to frequency-shift images taken at five different lateral- (Fig. 3b) and torsional-amplitude (Fig. 3c) setpoints. In general, the full width at half maximum (FWHM) of the peaks increases with decreasing amplitude setpoints (lateral/torsional). The histograms of the lateral frequency-shift images remain symmetric around a center value of approximately 120 Hz, whereas the histograms of the torsional frequency-shift images become asymmetric for torsional-amplitude setpoints smaller than 314 pm. As a result, the maxima of the histograms are shifted to more repulsive values. Moreover, the third eigenmode flexural frequency shift is only slightly influenced by the lateral- or torsional-eigenmode amplitude which corroborates the assumption of the independency of the in-plane (lateral/torsional) and the out-of-plane (flexural) eigenmodes (Fig. 3f,g (top graphs)). Interestingly, we observe that although the torsional- and lateral-amplitude setpoints are in most cases substantially larger than the interatomic spacing of the carbon atoms, we can still obtain atomic contrast in the frequency-shift images. To further investigate this phenomenon, we calculated the tip-trajectory resulting from the combined lateral-flexural (Fig. 4a,b) or torsional-flexural (Fig. 4c,d) oscillations of the cantilever according to equations (S1, S2) for two different lateral and torsional amplitudes, respectively.

It needs to be mentioned that in Fig. 4 only an extract of the full oscillation trajectory is shown. While imaging one position, almost every point in the area spanned by *A*_*flex*,2_ + *A*_*flex*,3_ and *A*_*tor,*1_ or *A*_*lat,*1_, respectively, is reached such as plotted in the Supplementary Information (Fig. S6). From Fig. 4 it becomes obvious that the interaction between tip and sample strongly depends on the curvature of the tip. For the situations shown in Fig. 4a,b,d we interpret, that the minimum distance from the lower turning point of the tip-trajectory to the intersection of the tip-trajectory and the horizontal line (purple dotted) defined by the interatomic spacing between two atoms (here 246 pm, due to the tip oscillating perpendicular to the carbon bonds) determines the indent (without distortion of the carbon atoms) of the tip into the carbon ring (vertical purple line). This indent without distortion of carbon atoms corresponds to the corrugation amplitude (*A*_*C-H*_). Thus, we defined the effective in-plane amplitudes as half of the horizontal distance between two carbon atoms (here: *A*_*lat,*1* (eff)*_ = *A*_*tor,*1 *(eff)*_ = 123 pm because the tip oscillates perpendicular to the carbon bonds) for the situations shown in Fig. 4a,b,d. From the situation shown in Fig. 4c we found that if the tip-trajectory shows a higher curvature, the effective amplitude is no longer equivalent to half of the distance between two carbon bonds. This is a result of the maximum possible corrugation amplitude which was reported by Kawai et al*.* to equal 85 pm in the repulsive regime^32^. This value was additionally verified by performing dynamic spectroscopy experiments such as shown in Fig. S5 of the Supplementary Information. Consequently, the effective amplitude is still determined by the intersection between the tip-trajectory and the horizontal line (purple dotted) defined by the interatomic spacing between two atoms, but it becomes smaller than 123 pm due to the limited corrugation amplitude. If we calculate the effective torsional amplitude from the tip-trajectory in Fig. 4c, assuming a corrugation amplitude of 85 pm, it would be approximately 83 pm, which is in line with the 79 pm determined in our recent work^4^. We can conclude that imaging the atomic structure of HOPG in the frequency-shift channel is also possible with in-plane oscillations larger than half of the interatomic spacing, but the resolution in the height images is strongly reduced due to the small corrugation amplitudes. Additionally, it needs to be considered that the frequency-shift images reflect the tip-sample interaction averaged over a few carbon hexagons. This needs to be kept in mind if forces are determined from the frequency-shift images. Nevertheless, due to the formation of plateaus in the torsional and lateral frequency shift values for larger in-plane oscillation amplitudes (see Fig. 3f,g (bottom graph)), we propose that conclusions about different interaction mechanisms, *i.e.* friction or shear, as a function of the different in-plane oscillation amplitudes can be drawn.

We point out that the most obvious way of determining the corrugation amplitude would be the analysis of the topography images. Unfortunately, the topography images are strongly influenced by the mean cantilever deflection, so that the corrugation amplitude is not directly accessible^4^.

### Calculation of lateral and torsional forces

**Figure 5:**
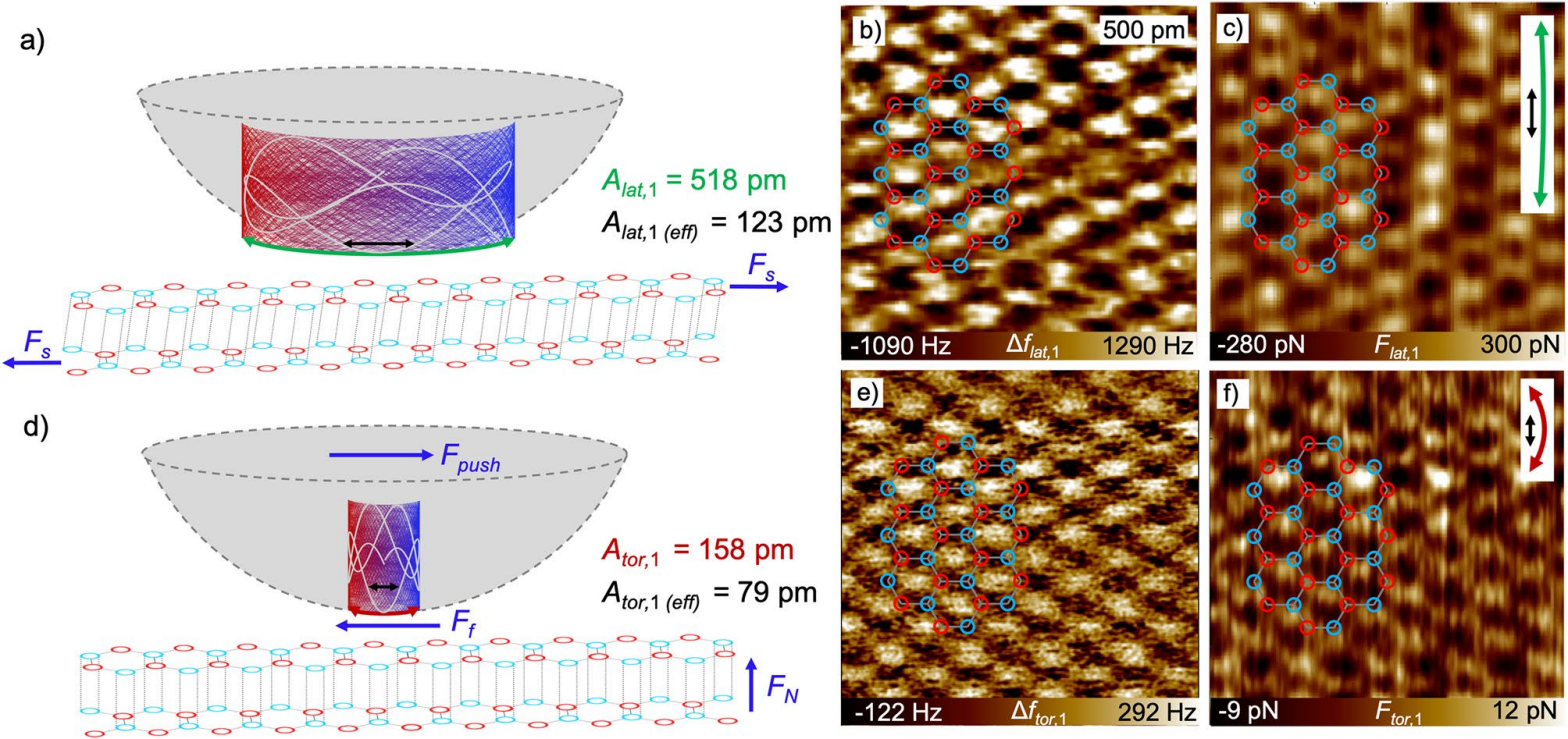
Scheme of the interaction between tip and HOPG surface for (**a**) a shearing interaction resulting from imaging in the AMFlex2-FMLat1-FMFlex3 mode with *A*_*lat,*1_ = 518 pm (*F*_*s*_: shear force) and (**d**) a frictional interaction resulting from imaging in the AMFlex2-FMTor1-FMFlex3 mode with *A*_*tor,*1_ = 158 pm, where *F*_*N*_ is the normal force acting perpendicular to the surface, *F*_*push*_ is the force originating from the movement of the tip in the direction of the in-plane oscillation and *F*_*f*_ is the frictional force counteracting *F*_*push*_ (*A*_*flex,*2_ = 110 pm and *A*_*flex,*3_ = 145 pm). (**b, e**) lateral and torsional frequency-shift images. (**c**,**f**) corresponding lateral and torsional force images calculated by the Fourier method and using the effective lateral/torsional amplitudes *A*_*lat,*1* (eff)*_ = 123 pm and *A*_*tor,*1* (eff)*_ = 79 pm.

From Fig. 3, we additionally observed that the full width at half maximum becomes much broader for the smaller lateral-amplitude setpoints compared to the torsional-amplitude setpoints. In order to identify the origin of the differences between torsional and lateral frequency-shift behavior, we calculated the forces from the images taken at *A*_*lat,*1_ = 518 pm and *A*_*tor,*1_ = 158 pm (*A*_*flex,*2_ = 110 pm and *A*_*flex,*3_ = 145 pm), respectively. The results are presented in Fig. 5. Schemes of the interaction between tip and sample are shown in Fig. 5a,d. The lateral and torsional frequency-shift images are shown in Fig. 5b,e. In Fig. 5c,f forces calculated from the frequency-shift images in Fig. 5b,e by using the Fourier method^4,35^ are shown.

Obviously, both, the torsional and the lateral force image in Fig. 5c,f reproduce the hexagonal structure of graphene. It needs to be mentioned that this is only the case if the forces are calculated by using the effective torsional or lateral amplitude. Interestingly, forces determined from the lateral frequency-shift image in Fig. 5b are around 50 times larger compared to the forces (Fig. 5f) calculated from the torsional frequency-shift images in Fig. 5e. We propose that this large difference in forces originates partially from the difference in force constants but also/mainly from different types of interaction mechanisms between tip and sample, i.e. friction and shear. While Weymouth et al*.*^11,36^ observed “non-contact friction” during imaging of an H-terminated Si(100) surface in dynamic lateral force microscopy using a qPlus sensor^13^, we assume that the lateral frequency shift determined on HOPG at 518 pm lateral amplitude mainly originates from a local shear due to relatively weak van-der-Waals interactions between the carbon layers. We assume this to happen for two reasons: First, the angle under which the tip touches the surface at the respective local position is less steep for the shown lateral oscillation compared to the torsional one. Second, the lateral stiffness of the cantilever is higher compared to the torsional stiffness which might lead to shear for a certain range of lateral oscillation amplitudes whereas torsional amplitudes of the same magnitude result in frictional interaction between tip and sample. In order to verify our assumption about shearing interaction for the constellation shown in Fig. 5a–c we calculated the lateral displacement *Δx* of the topmost carbon layer relative to the second one if we assume a shear modulus of 4.18 GPa in x-z-direction^37^. In a simple approximation, the shear modulus *G*_*xz*_ can be calculated by

1 $${G}_{xz}=\frac{{F}_{lat}\cdot d}{\Delta x\cdot {A}_{c}},$$

where *d* is the distance between two carbon layers (334 pm) and *A*_*c*_ is the contact area between tip and sample which can be calculated according to Hertz from the tip radius *R* and the indentation *δ*^38^

2 $${A}_{c}=\pi R\delta .$$

In order to determine the indentation *δ* which is caused by the tip oscillating over a carbon atom, we used Hertz contact mechanics model^38^

3 $$F=\frac{4}{3}{E}^{*}\sqrt{R{\delta }^{3}},$$

where E^*^ is the effective Young’s modulus which can be considered here as equal to the Young’s modulus *E*_zz_ of graphite due to the high stiffness of the diamond-like-carbon tip. The maximum value of *F*_*flex,*3_ was calculated in our resent work to be approximately 520 pN for the AMFlex2-FMTor1-FMFlex3 mode^4^. Due to the similarity of the third-eigenmode flexural frequency-shift values we chose this value as representative for all the other experiments. By rearranging Eq. (3) and inserting *E*_zz_ = 36.5 GPa as reported by Blakslee et al*.*^39^ we determined an indentation of approximately 50 pm. Using *δ* = 50 pm and rearranging Eq. (1), we calculated a local displacement of approximately *Δx* = 157 pm between the first and the second graphene layer. This implies a shift by the length of approximately one carbon–carbon bond (142 pm) which seems to be reasonable in distance. We corroborated our findings by performing additional friction force microscopy images, however, using a comparably large vertical deflection setpoint (43 nm ≙ 1100 nN) in order to ensure permanent contact between tip and sample. By means of the analysis of cross sections drawn through lateral deflection trace and retrace images, the actual lateral tip-position was calculated. The results are shown in Fig. S5 of the Supplementary Information. As expected, the typical stick–slip movement of the tip imaging an HOPG surface was observed^40^, however, the stiction was stretched out over a region of approximately 150 pm, which can be interpreted as a temporary shift of the topmost carbon layer relative to the second one.

For the tip-trajectory of the torsional oscillation shown in Fig. 5d, we observe a very similar oscillatory behavior, however, much more compact in the in-plane direction, resulting from the smaller torsional amplitude. As a consequence, the angle at which the tip approaches and finally touches the surface is much steeper and therefore a negligible shifting of the first carbon layer relative to the second is assumed to take place. We suppose that the torsional forces involved in this mechanism can be interpreted as strongly localized frictional forces which is on the one hand in accordance with the magnitude of frictional forces on graphene determined by Lee et al*.*^41^. On the other hand, the interpretation is in compliance with the observation of direction dependent torsional forces observed in our recent work^4^. Additionally, it needs to be highlighted, that the flexural/torsional oscillation of the cantilever simulated here is symmetric in out-of-plane direction but not in in-plane direction such as schematically shown in Fig. S6 of the Supplementary Information. This can explain the shift between topography and frequency-shift images such as shown in our recent work^4^. For the flexural/lateral oscillation the tip-trajectory is overall symmetric in both, in-plane and out-of-plane direction (see Fig. S6). Here, no shift between the height and the frequency-shift images could be observed. However, it needs to be mentioned that the resolution in the height images was strongly reduced if imaging was accomplished with the lateral instead of the torsional eigenmode. The effect can be attributed to the shear interaction between tip and sample. If the lateral or the torsional amplitudes become larger in size, we observe averaged friction along the surface which might explain the formation of the plateaus in the frequency shifts in Fig. 3f,g (bottom graph). Here, we also observed that the flexural frequency-shift values and therefore the flexural forces are only slightly influenced by the in-plane oscillation amplitudes. In theory, it should additionally be possible to determine the in-plane Young’s moduli *E*_*xx*_ = *E*_*yy*_ and *E*_*xy*_ by using higher torsional or lateral eigenmodes of the cantilever. Unfortunately, it is nontrivial to control these higher eigenmodes and determine their invOLS as well as the corresponding force constants. Nevertheless, this approach could be considered as an alternative method for the determination of the Young’s modulus of graphene instead of the frequently used techniques where graphene is spanned over membranes or holes and *E*_*xx*_ is calculated from nanoindentation experiments^42,43^. Therefore, we propose that the simultaneous determination of strongly localized in-plane and out-of-plane sample properties such as shear moduli and Young’s moduli using multifrequency AFM is feasible if very small amplitudes and adequate force constants are used for the analysis. The main advantage of determining elastic properties at the atomic scale is the possibility to locally assess the influence of atomic defects in the material, which were shown to strongly influence the mechanical stability of graphene samples on a larger scale^43^. Serving the large scientific interest to analyze the origin of friction anisotropy on graphene and graphite samples^44–47^, we are convinced that our presented multifrequency AFM method can substantially contribute to gain deeper insights into in-plane sample properties by comparing the mechanical properties on the atomic scale with that on the nano-/micrometer scale. The ability to compare material properties at different length scales in air under ambient conditions is of particular interest for the analysis of adsorbate formation^46,47^ or ripples^44,45^ on a graphitic surface which strongly influences the performance of graphene-based nanodevices. Additionally, the availability of torsional and lateral eigenmodes extends the spectrum of oscillation modes, facilitating the simultaneous acquisition of different in-plane sample properties, using one and the same cantilever.

## Conclusion

In summary, we demonstrated that performing multifrequency AFM with in-plane and out-of-plane components of the tip-motion has additional potential for atomic resolution imaging of HOPG surfaces. The coupling of the second flexural and the first torsional eigenmode facilitates imaging with atomic contrast at higher amplitude-setpoint ratios, although the quantitative interpretation of the results is challenging if the torsional and the lateral oscillation amplitude are simultaneously excited. In order to estimate the inverse optical lever sensitivity of the lateral cantilever eigenmode, a calibration procedure was proposed which is based on imaging a graphene wrinkle with different lateral amplitude setpoints while oscillating perpendicular to the wrinkle and comparing the images with the results of a Savitzky-Golay-filtered reference image. We compared the results from imaging in the AMFlex2-FMLat1-FMFlex3 and the AMFlex2-FMTor1-FMFlex3 mode. Analyzing the tip-trajectories and the in-plane forces resulting from both modes with the respective amplitudes, we proposed that imaging with small lateral amplitudes can result in localized shear of the first graphite layer with respect to the second one with a relative displacement of 157 pm. The applied out-of-plane forces caused an indentation of approximately 50 pm of the top graphene layer. Both values are in a reasonable range to propose that the AMFlex2-FMLat1-FMFlex3 method can be used for the simultaneous determination of *E*_*xz*_ = *E*_*yz*_ and *G*_*xz*_ = *G*_*yz*_. Imaging with the AMFlex2-FMTor1-FMFlex3 in contrast, promotes rather frictional forces which we mainly attributed to the smaller force constant and the curved tip-trajectory compared to the lateral eigenmode. Based on our findings, we suggest that the use of higher in-plane cantilever eigenmodes or generally stiffer cantilevers can provide a strategy to determine the shear moduli on HOPG and graphene also with torsional eigenmodes. The method carries great potential for future assessment of graphene in nanodevices where local differences in mechanical properties e.g. induced by defects or adsorbates, play a major role. To this end, the in-plane oscillation amplitudes need to be in the range of the interatomic spacings, which is still challenging to achieve while imaging in air under ambient conditions due to the small signal-to-noise ratio. With the presented method we aim to promote the analysis of friction anisotropy observed on graphene by the comparison of atomic and nano-/micrometer resolution images at different relative orientations between the hexagonal carbon lattice and the in-plane cantilever oscillation using torsional and/or lateral eigenmodes. This can help to investigate the origin of fundamental friction mechanisms underlaying graphene-based systems. Additionally, we expect that the presented method might not only be limited to the analysis of stiff samples but also can be applied for a broad range of materials, including soft matter, facilitating a complete in- and out-of-plane sample surface characterization.

## Materials and methods

### Sample

The HOPG sample (grade 2, mosaic spread angle: 0.8 ± 0.2°) was purchased from SPI Supplies (Structure Probe, Inc., West Chester, PA, USA). Cleaving was performed prior to the AFM experiments with adhesive tape in air under ambient conditions to expose a fresh clean surface.

### Cantilevers

Super-sharp cantilevers of the type HiResC15/Cr-Au purchased from Mikromasch (Innovative Solutions Bulgaria Ltd., Sofia, Bulgaria) were used. Although of the same type, the cantilevers showed slightly varying resonance frequencies resulting in “coupled” and “uncoupled” eigenmodes. For the AFM images shown in Fig. 2 we used a cantilever with “coupled” second flexural and first torsional eigenmodes as it depicted a close proximity of both resonance frequencies. For the AFM images shown in Figs. 3 and 5 cantilevers with “uncoupled” eigenmodes were used. The resonance frequencies, quality factors and force constants of the used cantilevers are listed in Table 1. Details for the determination of the force constants are given in Section 7 of the Supplementary Information. The tip radius of the cantilevers was *R* = 1 nm according to the manufacturer's data sheet.

**Table 1:** Resonance frequencies, quality factors and force constants of the “coupled” and “uncoupled” cantilevers HiResC15/Cr-Au.

HiResC15/Cr-Au	*f*_*0*_ in MHz			*Q*			*k* in N/m		
	“Coupled”	“Uncoupled”		“Coupled”	“Uncoupled”		“Uncoupled”		
		Tor	Lat		Tor	Lat	Tor	Lat	
1st flexural	0.274	0.266	0.263	606	594	585	26		25
2nd flexural	1.718	1.667	1.653	97	951	708	644		473
3rd flexural	*–*	4.615	4.562	–	381	296	3489		2406
1st torsional	1.735	1.730	1.720	1438	1341	1383	472		483
1st lateral	1.913	1.887	1.872	1546	1692	1634	1440		1395

The cantilever indexed by “Tor” was used for the images taken in AMFlex2-FMTor1-FMFlex3 mode and by “Lat” in the AMFlex2-FMLat1-FMFlex3 mode.

In Section 8 of the Supplementary Information we list the ratios between the flexural, torsional and lateral resonance frequencies determined for several cantilevers of the type HiResC15/Cr-Au for further considerations.

### Environmental conditions

The AFM-lab was equipped with a controlled ventilation system, which provided stable environmental conditions also inside the AFM chamber. The temperature and the relative humidity in the AFM chamber were tracked by sensors and remained stable during imaging (relative humidity: 22 ± 2%, temperature: 26 ± 2 °C).

### AFM setup

A Cypher S atomic force microscope (Asylum Research, Oxford Instruments, Santa Barbara, CA, USA) equipped with a built-in blueDrive photothermal excitation setup for dynamic AFM modes was used for all experiments. The blueDrive laser was focused at the fixed end of the cantilever and the lateral position was optimized by finding the spot that led to the maximum possible in-plane amplitude. A scheme for the approximate positioning of the laser spots on the cantilever is shown in Fig. S1e of the Supplementary Information. The respective frequency shifts (depending on the method used) were tracked with additional phase-locked loops (HF2PLL, Zurich Instruments, Zurich, Switzerland). The drive amplitudes of the frequency-modulated eigenmodes were adjusted in order to maintain constant amplitudes by two PID controllers implemented in the same instrument.

### Data processing

The topography and the torsional-amplitude error images were first-order flattened to remove any tilt from the images using the Igor Pro v6.36 software (WaveMetrics Inc., Lake Oswego, OR, USA). In order to remove noise from the small-scale images a 3 × 2 Gauss filter was applied to all AFM images except for the ones used for the determination of the lateral invOLS (Fig. S4). For the calculation of the torsional and the lateral force images in Fig. 5 we wrote a Matlab code (MATLAB R2018a, MathWorks Inc., Natick, MA, USA) for the implementation of the Fourier method based on the script provided by Seeholzer et al*.*^4,35^. The calculated force images were smoothed with a Savitzky–Golay filter over nine points with a first-order polynomial.

## Supplementary Information

Supplementary Information.

## Data Availability

All data will be made available from the authors upon reasonable request.
