# Supplementary material for: Torsional and lateral eigenmode oscillations for atomic resolution imaging of HOPG in air under ambient conditions
Source: Sci Rep. 2022 May 28;12:8981. doi: 10.1038/s41598-022-13065-9 (PMC9148301; doi:10.1038/s41598-022-13065-9)
Supplement: Supplementary file 1 — Supplementary Information. [file 41598_2022_13065_MOESM1_ESM.pdf]

Supplementary Information:
Torsional and lateral eigenmode oscillations for atomic resolution imaging of HOPG in air under ambient conditions

Anna L. Eichhorn[1] and Christian Dietz[1]*

[1]Physics of Surfaces, Institute of Materials Science, Technische Universität Darmstadt, Alarich-Weiss-Str. 2, 64287 Darmstadt, Germany

Email: eichhorn@pos.tu-darmstadt.de, dietz@pos.tu-darmstadt.de

1. Illustration of in-plane oscillation optical detection

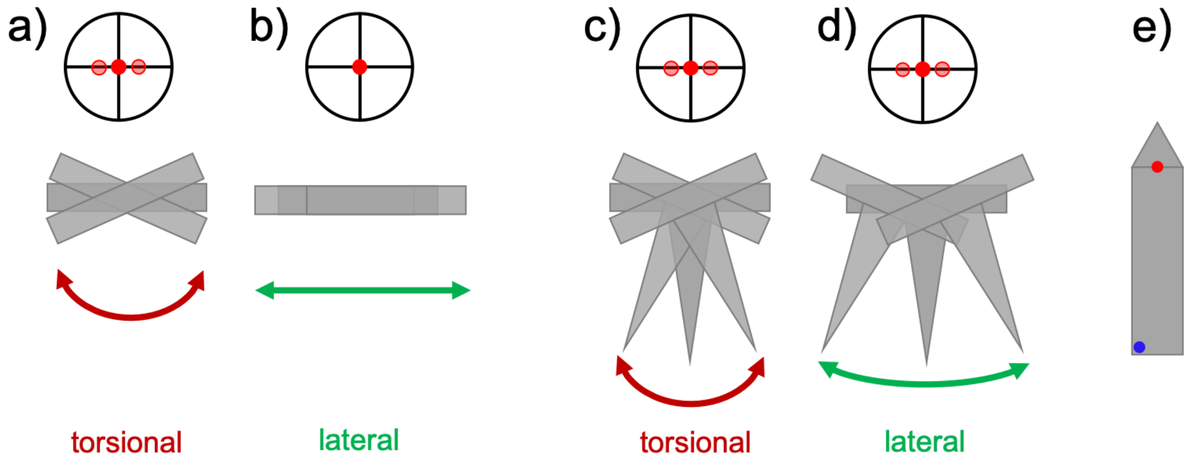

Figure S1: Illustration of a torsional (a, c) and a lateral eigenmode oscillation (b, d) for tip-less (a, b) and tip-containing cantilevers (c, d) and scheme of the detection (red) and photothermal excitation laser (blue) (e). Due to the rotational motion induced by the torsional eigenmode oscillation, the amplitude can be optically detected on a segmented photodiode for tip-less (a) and tip-containing cantilevers (c) in the same way. On the other hand, it is not possible to detect the lateral oscillation of a tip-less cantilever (b) by means of the standard optical beam detection technique. Nevertheless, as explained by Ding et al.[1], due to the small torsional motion induced by the tip attached to the cantilever, the lateral oscillation can be detected using the standard optical beam detection technique. To determine the optical lever sensitivity, additional considerations are required as discussed in detail in section 4 "Calibration of lateral oscillation amplitude inverse optical lever sensitivity" of this Supplementary Information.

2. Estimation of tip-trajectory from in-plane and out-of-plane deflection

The cantilever oscillation in out-of-plane (z) and in-plane (x) direction can be estimated by linear combinations of cosine functions including the amplitude setpoints A_i, the resonance frequencies $f_{0(i)}$, the time t and the phase Φ_{AM} of the oscillation used for the topographic feedback in amplitude modulation[2]. The static components z_0 and x_0 have been neglected for simplicity. For the calculations behind the figures of the tip-trajectories shown in the main text, equations (S1, S2) were used

$$z(t) = z_0 + z_{flex,2}(t) + z_{flex,3}(t) \approx A_{flex,2}\cos\left(2\pi f_{0\,(flex,2)}t - \Phi_{flex,2}\right) + A_{flex,3}\cos\left(2\pi f_{0\,(flex,3)}t - \Phi_{flex,3}\right), \quad \text{(S1)}$$

$$x(t) = x_0 + x_{tor}(t) + x_{lat}(t) \approx A_{tor}\cos\left(2\pi f_{0\,(tor)}t - \Phi_{tor}\right) + A_{lat}\cos\left(2\pi f_{0\,(lat)}t - \Phi_{lat}\right). \quad \text{(S2)}$$

If the respective channel is frequency modulated, the phase equals $\pi/2$. For the tip-trajectories shown for the AMFlex2-OLTor1-FMLat1 mode, the phase of the torsional oscillation was assumed to be 90° due to the lack of channels which could be detected simultaneously. In order to analyze the influence of the torsional phase, we simulated tip-trajectories with $\Phi_{tor,1}$ = 30°, 45°, 60°, 90°, 100° and 120° as shown in Figure S2.

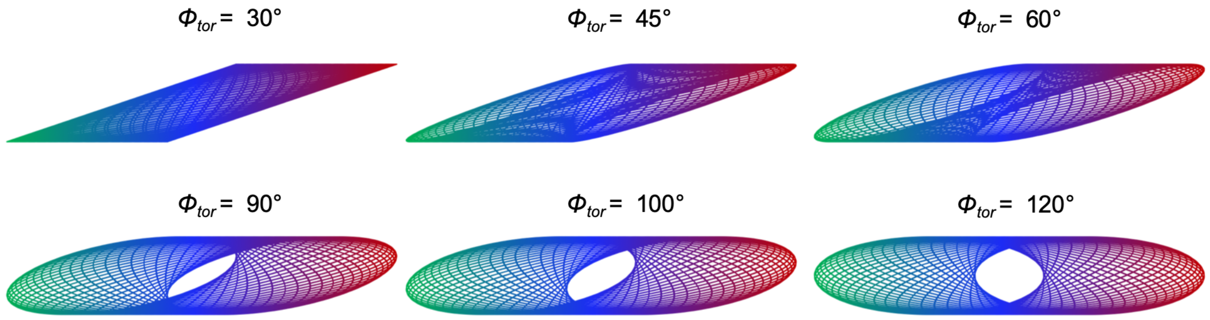

Figure S2: Tip-trajectories calculated using equations S1 and S2 for a "coupled" cantilever (resonance frequencies listed in Table 1 of the main text) oscillating in the AMFlex2-OLTor1-FMLat1 mode with $A_{flex,2}$ = 700 pm, $A_{tor,1}$ = 1232 pm and $A_{lat,1}$ = 863 pm for different torsional phases.

From Figure S2 we can see that the torsional phase has a significant influence on the tip-trajectory which complicates the interpretation of the measurement data taken in the AMFlex2-OLTor1-FMLat1 mode. Consequently, we suggest to focus on imaging with "uncoupled" cantilevers if quantification is required.

3. Comparison of atomic resolution imaging on HOPG in the repulsive and the attractive regime

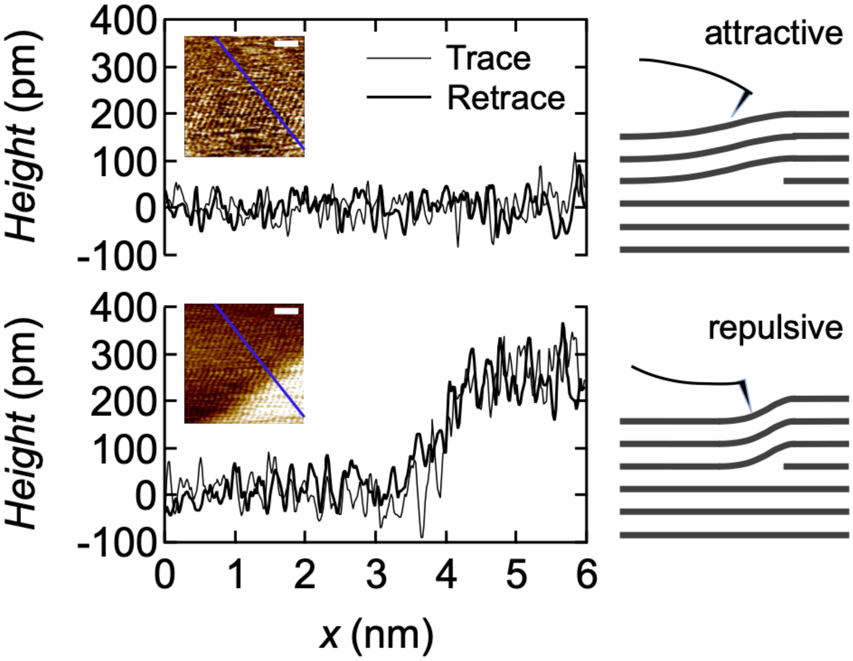

Figure S3: Cross sections through height images of HOPG at two different *z*-sensor positions and schemes of the alleged tip-sample interaction at the step edge in the attractive and the repulsive regime. The insets show the height images (retrace) of Figure 2j at Figure 2j at $z \approx 3.1$ nm (top) and Figure 2k at $z \approx 3.7$ nm (bottom). The position of the cross sections is marked in blue. The thin black cross sections are drawn through the trace images (not shown) and the thicker cross sections through the retrace images at the same position. Scale bar: 400 pm.

4. Calibration of lateral oscillation amplitude inverse optical lever sensitivity

The lateral cantilever oscillation is rarely used for AFM imaging because of the difficulties to calibrate its inverse optical lever sensitivity (invOLS). As discussed in detail by Ding et al.[1] the lateral oscillation itself cannot be detected by the photodiode. But due to the tip attached to the cantilever, a small torsion is induced which results in a measurable movement of the reflected laser beam on the photodiode, that allows for the detection of the lateral resonance by the help of a thermal spectra or cantilever tunes without being in contact with the sample. While the torsional oscillation amplitude invOLS can be determined e.g. from thermal noise spectra[15], there is to the best of our knowledge currently no method for the lateral oscillation invOLS calibration. Here, we present an approach based on imaging a graphene wrinkle on an HOPG sample with different lateral-oscillation-amplitude setpoints in AMFlex2-OLTor1-FMLat1 mode. The idea behind this method is, that the effect of an increasing lateral oscillation amplitude can be compared to a smoothening of imaging data points. In Figure S4a and f we show the averaged cross sections (20 pixel) through the height images (b-e) and the lateral frequency-shift images (g-j) taken at different lateral-oscillation-amplitude setpoints. All images were taken at a constant second flexural-eigenmode amplitude of 238 pm and at a scan angle of 90° resulting in both, fast scan direction and lateral oscillation direction being perpendicular to the graphene wrinkle. The orange (b,g)/black (c,h)/blue (d,i)/red (e,j) marked cross sections result from the imaging data taken at lateral-oscillation-amplitude setpoints of 5 mV/10 mV/20 mV/40 mV. The black/blue/red dotted lines in Figure S4a and f result from smoothening of the orange curves ($A_{lat,1}$ = 5 mV) over 23/45/89 pixel and multiplication (height)/division ($\Delta f_{lat,1}$) by the weighing factors 1.25/1.85/2.80 which we introduced in order to optimize the fit. The wheighing factors $w_{lat,1}$ can be determined according to equation (S3) which was found empirically

$$w_{lat,1} = 1.5^{\frac{A_{lat,1(large)}}{2A_{lat,1(small)}}}. \tag{S3}$$

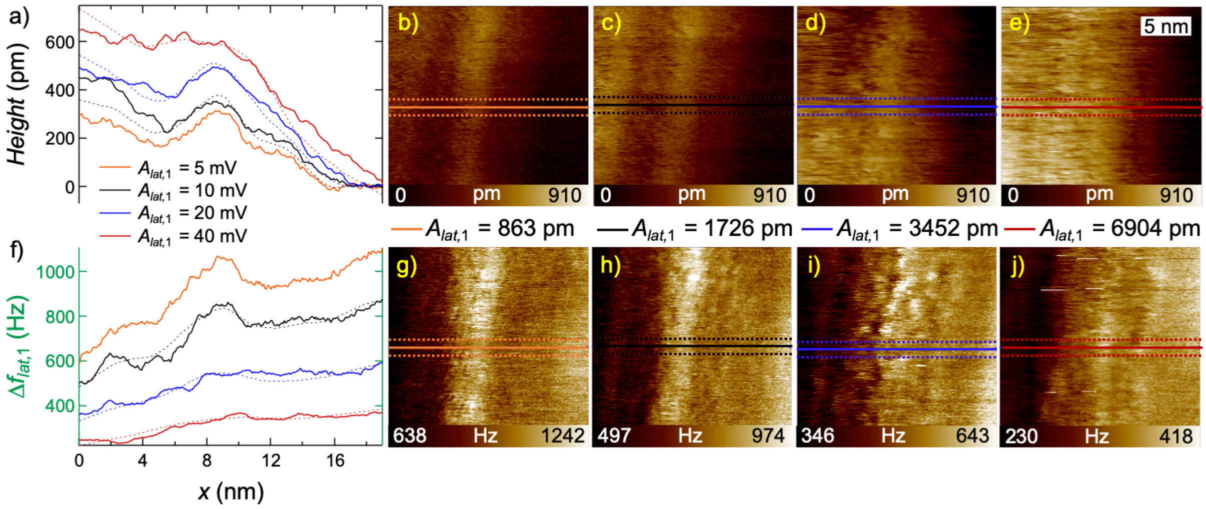

Figure S4: Calibration of lateral-oscillation-eigenmode amplitude at a graphene wrinkle on HOPG. The solid lines in a) and f) show the height and the lateral frequency shift for different lateral-eigenmode setpoints along the averaged cross sections (20 pixel) marked in b-e) and g-j). The images were taken in the AMFlex2-OLTor1-FMLat1 mode with a free second flexural-eigenmode amplitude of 765 pm and a setpoint of 238 pm. The scan angle was 90° so that both, the fast scan direction and the direction of the lateral oscillation were orineted perpendicular to the wrinkle. The dotted black/blue/red lines in a) and f) represent the orange curve smoothed over 23/45/89 pixel and multiplication (height)/division ($\Delta f_{lat,1}$) by the weighing factors 1.25/1.85/2.80 which we introduced in order to optimize the fit.

From the cross sections in Figure S4a and f and from the corresponding images in Figure S4b-e and g-j we can observe that the increase in $A_{lat,1}$ from 5 mV to 40 mV leads to stronger lateral averaging of the height and the lateral frequency-shift values. If we compare e.g. the orange ($A_{lat,1}$ = 5 mV) with the red curve ($A_{lat,1}$ = 40 mV) in Figure S4a we can directly see that for the smaller $A_{lat,1}$ two hillocks can be resolved whereas for the larger $A_{lat,1}$ only one broader hillock is visible. In Figure S4f the lateral frequency shift shows a clearly visible hillock for $A_{lat,1}$ = 5 mV (orange) whereas for $A_{lat,1}$ = 40 mV(red) the hillock is hardly visible. Based on these observations we assumed that the cross sections of the images taken with $A_{lat,1}$ = 10 mV, $A_{lat,1}$ = 20 mV and $A_{lat,1}$ = 40 mV can be reproduced by smoothening of the orange curve which represents the cross sectional data resulting from $A_{lat,1}$ = 5 mV. By smoothening the orange curves in Figure S4a and f with a Savitzky-Golay filter of first grade over 23, 45 and 89 pixel, respectively, we were able to reproduce the general trend of the black, blue and red curves with satisfying accuracy. After treating the smoothed averaged cross-sections with the appropriate weighing factors, the black, blue and red dotted curves shown in Figure S4a and f were gained. Although there are small deviations between the dotted and the solid curves in Figure S4a, in

particular at the end and the beginning of the cross sections, the overall trend is nicely reproduced. From the number of pixel over which the smoothening was performed, we determined the optical invOLS of the lateral oscillation amplitude according to equations (S4, S5)

$$\left(A_{lat,1} - A_{lat,1\,(ref)}\right)[\mathrm{nm}] = \frac{l}{n_{line}} \frac{n_{smooth} - 1}{2}, \tag{S4}$$

$$s_{lat,1} = \frac{\left(A_{lat,1} - A_{lat,1\,(ref)}\right)[\mathrm{nm}]}{\left(A_{lat,1} - A_{lat,1\,(ref)}\right)[\mathrm{V}]}, \tag{S5}$$

where $A_{lat,1\,(ref)}$ is the lateral-amplitude setpoint which was used for the reference image and the corresponding cross section chosen to be smoothened (here: $A_{lat,1\,(ref)}$ = 5 mV, orange line). In our case the scan size (l) was 20 nm and the number of pixel/line (n_{line}) was 256. Due to the definition of the amplitude as half of the peak-to-peak value, the number of pixel over which was smoothed (n_{smooth}) needs to be devided by two after one pixel was substracted (the pixel at the center position). The determination of the optical invOLS was done by comparing the differences of the lateral amplitudes in nanometers and in volts such as shown in equation (S5). Following the explained procedure, we determined a lateral sensitivity of 122 nm/V. The resulting lateral-oscillation-amplitude setpoints in nanometers corresponding to the images in Figure S4 are shown in the center row. Consequently, the invOLS of the lateral oscillation is around eleven times larger compared to the invOLS of the torsional oscillation which we determined in our recent work to be 11 nm/V[4]. At first glance this seems to be counterintuitive because the lateral stiffness is higher than the torsional stiffness for the type of cantilever used here (see Section 7 "Calibration of inverse optical lever sensitivities and force constants" of this Supporting Information), which should also result in a higher optical lever sensitivity (lower invOLS) for the lateral oscillation. However, if we take into account that the signal on the photodiode cannot sense the lateral but only the torsional component of the lateral oscillation induced by the tip, it is reasonable to believe that the invOLS is larger for the lateral eigenmode oscillation.

5. Vertical and lateral tip position estimated from dynamic spectroscopy and friction force microscopy (FFM)

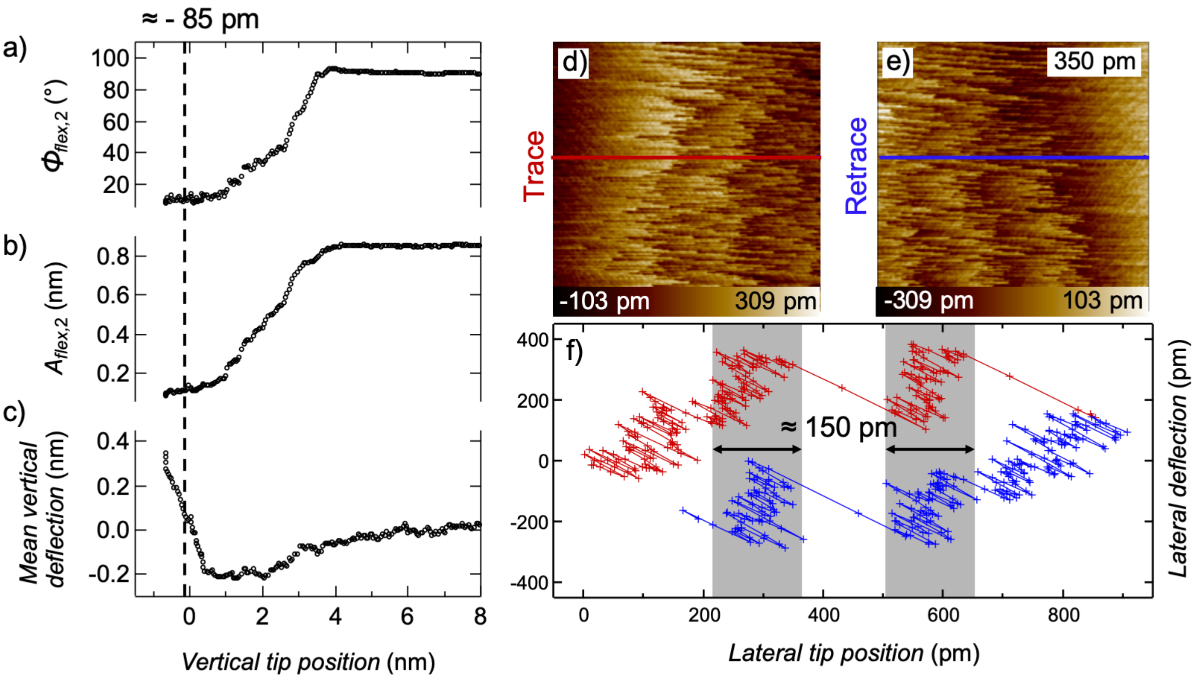

Figure S5: Vertical and lateral tip position estimated from dynamic spectroscopy (a-c) and friction force microscopy (FFM) (d-f), respectively. Second flexural phase (a), amplitude (b) and mean vertical deflection (c) as a function of the vertical tip position (*z*-position corrected for vertical deflection). The dotted line at approximately -85 pm indicates the vertical tip position, where the second flexural eigenmode setpoint of 110 pm was reached. This corroborates the estimation of an indentation depth of 14-85 pm while imaging an HOPG surface. Nevertheless, it was not possible to determine differences in indentation depth when imaging the mean vertical deflection while varying the lateral or torsional oscillation amplitude. From a and b it becomes clear that the second flexural phase and amplitude form plateaus close to the HOPG surface. We assume that this is more likely a consequence of oscillating inside natural water layers covering the HOPG surface rather than a permanent contact between tip and sample. This would imply imaging in contact resonance mode where the frequency shift is expected to be much larger compared to the values observed in our study. In d and e, the trace and retrace lateral deflection images of an HOPG surface analyzed with friction force microscopy are shown, respectively. It needs to be mentioned that the vertical deflection setpoint (1100 nN) was much larger compared to the mean vertical deflection values reached in dynamic spectroscopy because no stable contact was achieved using smaller vertical deflection values. The horizontal lines indicate the positions at which the cross sections shown in f were taken, where the abscissa was selected as the lateral tip position, calculated from the

x-position of the cross section corrected for the lateral tip-deflection. The calibration of the in-plane static sensitivity was performed on a silicon substrate, following the protocol of Dietz[3]. From the graph in f, on the one hand, the typical stick-slip motion (gray-white) can be observed. On the other hand, it becomes evident, that the tip-position was not completely fixed within the gray marked area, but that the tip showed an overall movement of around 150 pm. This observation can be interpreted as a corroboration of the observations from Figure 4, that while imaging, a local relative displacement of around 150 pm between two carbon layers could be induced.

6. Comparison of tip trajectories in AMFlex2-FMLat1-FMFlex3 and AMFlex2-FMTor1-FMFlex3 mode

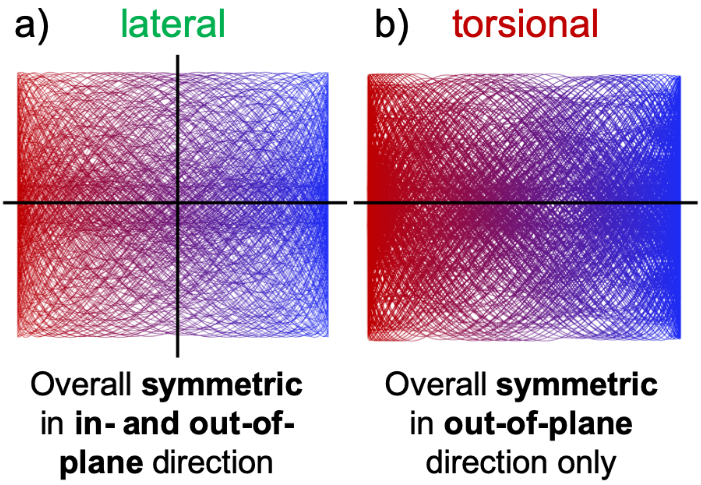

Figure S6: Comparison of the tip-trajectory resulting from (a) the AMFlex2-FMLat1-FMFlex3 mode and (b) the AMFlex2-FMTor1-FMFlex3 with $A_{flex,2} = 110$ pm, $A_{flex,3} = 145$ pm and $A_{lat,1} = A_{tor,1} = 328$ pm.

7. Calibration of inverse optical lever sensitivities and force constants

The determination of the inverse optical lever sensitivities (invOLS) as well as the force constants of the flexural and the torsional eigenmodes were described in detail in our recent paper[4]. A method for the determination of the lateral invOLS was provided in Section 4 of this Supplementary Information. The lateral force constant was calculated from the first flexural force constant, the width b and the thickness t of the cantilever[5]

$$k_{lat,1} = k_{flex,1}\left(\frac{b}{t}\right)^2. \tag{S3}$$

8. Comparison of resonance frequencies

Table 1: Averaged resonance-frequency ratios and standard deviations determined for different amounts of cantilevers (a: 18, b: 6, c: 17) of the type HiResC15/Cr-Au. The relative deviation, relating the standard deviation to the average value is shown in percentage. The last row includes the theoretical relations between the different resonance frequencies for rectangular cantilevers, where L is the length, b is the width and t is the thickness of the cantilever.[6,7]

$f_{0\,(flex,2)}/f_{0\,(flex,1)}{}^{a}$	$f_{0\,(flex,3)}/f_{0\,(flex,1)}{}^{b}$	$f_{0\,(tor,1)}/f_{0\,(flex,2)}{}^{a}$	$f_{0\,(lat,1)}/f_{0\,(flex,2)}{}^{c}$	$f_{0\,(lat,1)}/f_{0\,(tor,1)}{}^{c}$
6.29 ± 0.01	17.40 ± 0.04	1.04 ± 0.03	1.14 ± 0.02	1.09 ± 0.01
0.2 %	0.2 %	2.6 %	2.1 %	0.5 %
= 6.27	= 17.55	$\propto L/t$	$\propto b/t$	$\propto b/L$

From Table 1 we can see that the relations between the different flexural eigenmodes are very close to the theoretical values for rectangular cantilevers[6] and show only a small relative deviation. Additionally, we found that the relations between the resonance frequencies of the torsional, lateral and flexural eigenmodes were also comparably stable, although slightly larger relative deviations were observed, especially for the ratios of torsional to flexural (2.6 %) and lateral to flexural resonance frequencies (2.1 %). From theoretical considerations presented by Young et al.[7] regarding the dependency of the resonance frequencies on the cantilever dimensions, we concluded that the slightly larger spreading of the relation between torsional/flexural and lateral/flexural resonance frequencies can most likely be attributed to small variations in the thickness of the cantilevers. Due to the fact that the relation between the torsional and the second flexural resonance frequency is close to zero, even the small relative deviation of 2.6 % leads to the occurrence of "coupled" and "uncoupled" cantilevers as described in the main text.

References

1 Ding, R.-F., Yang, C.-W., Huang, K.-Y. & Hwang, I.-S. High-sensitivity imaging with lateral resonance mode atomic force microscopy. *Nanoscale* **8**, 18421-18427, doi:10.1039/C6NR04151E (2016).

2 Benaglia, S., Amo, C. A. & Garcia, R. Fast, quantitative and high resolution mapping of viscoelastic properties with bimodal AFM. *Nanoscale* **11**, 15289-15297, doi:10.1039/c9nr04396a (2019).

3 Dietz, C. Sensing in-plane nanomechanical surface and sub-surface properties of polymers: local shear stress as function of the indentation depth. *Nanoscale* **10**, 460-468, doi:10.1039/c7nr07147g (2018).

4 Eichhorn, A. L. & Dietz, C. Simultaneous Deconvolution of In-Plane and Out-of-Plane Forces of HOPG at the Atomic Scale under Ambient Conditions by Multifrequency Atomic Force Microscopy. *Advanced Materials Interfaces* **8**, doi:10.1002/admi.202101288 (2021).

5 Mullin, N. & Hobbs, J. K. A non-contact, thermal noise based method for the calibration of lateral deflection sensitivity in atomic force microscopy. *Review of Scientific Instruments* **85**, 113703, doi:10.1063/1.4901221 (2014).

6 Garcia, R. & Herruzo, E. T. The emergence of multifrequency force microscopy. *Nature Nanotechnology* **7**, 217-226, doi:10.1038/nnano.2012.38 (2012).

7 Young, W. C. & Budynas, R. G. *Roark's Formulas for Stress and Strain*. 7th edn, (McGraw-Hill, 2002).
